# “BEYOND JUST A SINGING GROUP”: PERCEPTIONS OF PARTICIPANTS, CAREGIVERS, AND LEADERS OF PARKINSON’S SINGING GROUPS

**DOI:** 10.1093/geroni/igad104.2936

**Published:** 2023-12-21

**Authors:** Jessy Brown, Cailie Logue, Maya Bartel, Tera Jordan, Elizabeth Stegemoller

**Affiliations:** Iowa State University, Ames, Iowa, United States; Iowa State University, Ames, Iowa, United States; Iowa State University, Ames, Iowa, United States; Iowa State University, Ames, Iowa, United States; Iowa State University, Ames, Iowa, United States

## Abstract

Parkinson’s disease (PD) is the second most prevalent neurodegenerative disease after Alzheimer’s disease, and its prevalence is increasing. There is no cure, so symptom treatment is currently the only method to decrease the disease burden for individuals diagnosed with PD. A common treatment modality option that has been shown to improve both motor and non-motor symptoms in PD is therapeutic singing, often in a group setting. Previous literature has explored perceptions of singing groups for persons with PD (PwPD) and their caregivers (CGs) and has found overall positive experiences and outcomes. No study has sought to understand the perspectives of the leaders of the singing groups. The purpose of this study was to understand the perceptions and experiences of participants in singing groups (n=31), their caregivers (n=8), and those leading the groups (n=10) across several different singing groups in the United States. A qualitative phenomenological methodological framework was used with principles of grounded theory to make meaning from the transcripts. Three main themes emerged from the data: peer support, proactive PD management, and the effects of the pandemic. These findings indicate that even during a global pandemic, all three participant groups experienced positive outcomes resulting from peer support and either experiencing or witnessing effective management of PD symptoms. All three participant groups reported similar perceptions and experiences about their involvement which reinforces the rationale to use singing as a treatment modality for PD.

